# Effect of nickel, cobalt, and iron on methanogenesis from methanol and cometabolic conversion of 1,2‐dichloroethene by *Methanosarcina barkeri*

**DOI:** 10.1002/bab.1925

**Published:** 2020-05-12

**Authors:** Lara M. Paulo, Mohamad R. Hidayat, Giulio Moretti, Alfons J. M. Stams, Diana Z. Sousa

**Affiliations:** ^1^ Laboratory of Microbiology Wageningen University & Research Wageningen The Netherlands; ^2^ Laboratory of Microbiology MESVA Department University of L'Aquila Via Vetoio Coppito (AQ) Italy

**Keywords:** cometabolic dechlorination, metals, methanogenesis

## Abstract

Methanogens are responsible for the last step in anaerobic digestion (AD), in which methane (a biofuel) is produced. Some methanogens can cometabolize chlorinated pollutants, contributing for their removal during AD. Methanogenic cofactors involved in cometabolic reductive dechlorination, such as F_430_ and cobalamin, contain metal ions (nickel, cobalt, iron) in their structure. We hypothesized that the supplementation of trace metals could improve methane production and the cometabolic dechlorination of 1,2‐dichloroethene (DCE) by pure cultures of *Methanosarcina barkeri*. Nickel, cobalt, and iron were added to cultures of *M. barkeri* growing on methanol and methanol plus DCE. Metal amendment improved DCE dechlorination to vinyl chloride (VC): assays with 20 µM of Fe^3+^ showed the highest final concentration of VC (5× higher than in controls without Fe^3+^), but also in assays with 5.5 µM of Co^2+^ and 5 µM of Ni^2+^ VC formation was improved (3.5–4× higher than in controls without the respective metals). Dosing of metals could be useful to improve anaerobic removal of chlorinated compounds, and more importantly decrease the detrimental effect of DCE on methane production in anaerobic digesters.

## Graphical abstract

*Methanosarcina barkeri* (DSM800^T^) is able to cometabolically reduce 1,2‐dichloroethene (DCE) to vinyl chloride (VC) with methanol as electron donor. However, the presence of DCE inhibits methane production by this organism. In this study, we evaluate if the supplementation of cobalt, nickel, and iron (required for methanogenic activity and reductive dechlorination) can improve methane production by *M. barkeri* in the presence of DCE.

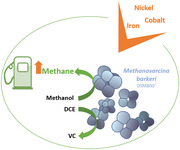

**Abbreviations**
AD: anaerobic digestion
DCE: 1,2‐dichloroethene
GC‐FID: gas chromatography system with flame ionization detector
HPLC: high performace liquid chromatographhy system
PCE: tetrachloroethene
TCE: trichloroethene
VC: Vinyl chloride

## 1 Introduction

Tetrachloroethene (PCE), trichloroethene (TCE), and other chlorinated compounds are extensively used in industry (e.g., as solvents, extractants, or degreasing agents) and, therefore, commonly found in wastewaters [1, 2, 3]. Partial dechlorination of these compounds also results in the occurrence of lesser chlorinated ethenes, such as *cis/trans*‐1,2‐dichloroethene (DCE) and vinyl chloride (VC), as secondary pollutants [1, 4,5]. Highly chlorinated compounds, such as PCE, are resistant to aerobic degradation [6], but can be cometabolized or used as terminal electron acceptor by a diversity of anaerobic microorganisms (including nitrate and sulfate reducers, acetogens, and methanogens) [6, 7]. Anaerobic treatment of wastewaters with chlorinated compounds would also eliminate pollution associated with their volatilization, often observed in conventional wastewaters aerobic treatment plants; moreover, anaerobic digestion results in the production of methane‐containing biogas, a valuable product that can be used as biofuel. Methane‐producing archaea, methanogens, are key players in anaerobic digesters [8] and are also known to contribute to the dechlorination processes in these systems [9, 10]. *Methanosarcina* species are reported to have dechlorinating activity; for example, *Methanosarcina barkeri* is described to be able to reduce dichloroethane [10], whereas *M. thermophila* can dechlorinate PCE [11]. We recently detected microorganisms closely related to *M. barkeri* in enrichment cultures converting DCE using methanol or H_2_/CO_2_ as substrates [12]. Despite the fact that they were not the most abundant archaea, they were the only microorganism found in all the enrichment conditions, independent from the source of inocula.

Corrinoids, porphyrins, and cofactors are key factors for methane production as they are required for most of the enzymes in the methanogenic pathway. Moreover, they can also play an important role in cometabolic dechlorination. For example, it was observed that cobalamin and cofactor F_430_ from *M. barkeri* can reductively dechlorinated 1,2‐dichloroethane (1,2‐DCA) to ethene or chloroethane (CA) [13]. Furthermore, it was observed that vitamin B_12_, cofactor F_430_, and hematin could catalyze the reductive dechlorination of polychlorinated ethylenes and benzenes, but ferredoxins and azurin could not [14]. Vitamin B_12_ and cofactor F_430_ could sequentially catalyze dechlorination of PCE to ethene, but hematin only led to the dechlorination until VC. Jablonski and Ferry observed that the corrinoid‐containing carbon monoxide dehydrogenase of *M. thermophila* was able to degrade TCE to *cis‐*, *trans‐*, and 1,1‐DCE, VC, and ethene [15].

Most of these porphyrins, corrinoids, and cofactors have metals, typically cobalt (Co), nickel (Ni), and iron (Fe) in their structure. These metals have a fundamental role in methanogenesis and, as a consequence, in biogas production. For example, Ni and Fe are present in Ni–Fe hydrogenases. Ni is also present in cofactor F_430_, and Fe is required for most redox enzymes involved in methanogenesis. Co is present in cobalamides that have an important role as methyl carriers in methanogenesis from methylated compounds. Moreover, cobalamides are also described to be intermediates between methyl‐H_4_MPT and coenzyme M [16]. Metal supplementation has proven to be a good strategy to improve several biological processes involving enzymes that require metal ions, including methane production during anaerobic digestion of organic waste [17, 18, 19, 20, 21] and reductive dechlorination [22]. Furthermore, in mixed dechlorinating cultures, it is known that methanogenic cofactors can be scavanged by dechlorinating bacteria that are unable to synthesize them [5, 23]. For example, it is reported that *Dehalococcoides* can benefit from the presence of methanogens in low abundace to provide vitamin B12 and other cofactors required for the reductive dehalogenase enzymes [23]. Metal supplementation can then indirectly improve decholorination rates.

In this work, the effect of supplementation of metal ions Fe^3+^, Ni^2+^, and Co^2+^, which are required for methanogenic activity, but also for cometabolic reduction of DCE, was studied in pure cultures of *M. barkeri* (DSM 800). The aim was to assess the impact of different metals and their concentrations on methane production in the presence and absence of DCE. Furthermore, it was evaluated if both methanogenesis and reductive dechlorination could be improved, and if the last could be improved in a way that mitigated the inhibitory effects of the chlorinated compound on methane production.

**Highlights**

Chlorinated compounds often affect the production of methane (biofuel) in anaerobic digesters. Here we studied the effect of supplementing metals (cobalt, nickel, and iron) in the performance of *Methanosarcina barkeri*, a methanogen that cometabolizes dichloroethene (DCE). Cobalt, nickel, and iron are present in enzymes and cofactors involved in methanogenesis and reductive dechlorination. Overall, metal addition had a positive effect in methanogenesis from methanol by *M. barkeri*. Addition of nickel and iron diminished the detrimental effect of DCE on methane production.

## 2 Materials and Methods

### 2.1 Source of microorganism and media composition

An axenic active culture of *M. barkeri* (DSM 800^T^) was ordered from the German culture collection (DSMZ, Braunschweig, Germany). Mineral‐basal medium was prepared according to the protocol previously described by Stams et al. [24] and supplemented with 5 mg/L of yeast extract. Forty‐five milliliters of mineral medium was dispensed into 120 mL serum bottles that were sealed with rubber stoppers and aluminum cramp caps. Bottles’ headspace was flushed with N_2_/CO_2_ (80:20; % v/v; 1.5 atm).

### 2.2 Dechlorination assays and effect of trace metals

Dechlorination assays with *M. barkeri* were done using 125 mM of methanol as electron donor and 0.5 mM DCE. Controls without DCE were also included. To test the effect of metals on the dechlorination of DCE, tests were performed with NiCl_2_ (5, 10, 15, and 20 µM), CoCl_2_ (5.5, 10.5, 15.5, and 20.5 µM), and FeCl_3_ (22, 27, 32, and 37 µM). A metal concentration range was selected based on values described by others as reviewed by Paulo et al. [25]. Controls with DCE but without metal addition were included. Bottles were inoculated with 2.5 mL of exponentially grown culture of *M. barkeri* and incubated at 30 °C in the dark for 336 H. The assays were performed in triplicate.

### 2.3 Analytical methods and calculations

Bottles’ headspace was analyzed for H_2_ and CH_4_ with a Compact GC^4.0^ (Interscience, Breda, The Netherlands) equipped with Carboxen 1010 PLOT column (30 m × 0.32 mm) (Merck KGaA, Darmstadt, Germany) followed by a Molsieve 5A column (30 m × 0.32 mm) (Restek GmbH, Bad Homburg, Germany), with argon as carrier gas at 0.8 mL Min^−1^, and a thermal conductivity detector. The temperature of the valve (injection) oven was kept at 60 °C, while column and detector were kept at 100 °C. The initial methane production rate was determined for each test as the initial slope of the curve (as exemplified in Figure S.1 in the Supporting Information) obtained by plotting the measured methane in the triplicate assays.

Concentrations of DCE and VC were determined by analyzing the bottles’ headspace on a Varian GC‐FID (CP‐3800) (Thermo Scientific) equipped with a split–splitless injector followed by an RT‐Q Bond column (30 m × 0.32 mm) (Restek GmbH, Bad Homburg, Germany). Gas carrier was helium at a flow of 2 mL Min^−1^. Temperature was set to 40 °C for 1 Min, followed by a temperature ramp up to 200 °C in 4 Min and additional 5 Min at 200 °C. Standards for chlorinated compounds were prepared by adding a known amount of chromatography‐grade DCE and VC to a serum bottle with the same headspace to liquid ratio as the assay bottles. All the vials (including the standards) were kept under controlled temperature in a thermal‐bath to guarantee that the conditions were the same during the analysis.

Liquid samples were analyzed for volatile fatty acids and alcohols with an Acella HPLC (Thermo Scientific, Waltham, MA) equipped with a Varian Metacarb 67H column (300 × 6.5 mm) (Agilent, Santa Clara, MA) and a refractive index detector. Column was kept at 45 °C and running with 0.01 N of H_2_SO_4_ as eluent at a flow rate of 0.8 mL Min^−1^.

Fe^2+^ and Fe^3+^ were determined with the ferrozine method [26]. In short, the ferrozine reagent reacts with the Fe^2+^ to form a stable magenta complex and the maximum absorbance is recorded at 562 nm. A reducing agent is then added to reduce all Fe^3+^ to Fe^2+^, given the total iron concentration in the sample. The absorbance is then recorded at the same wavelength. The concentration of Fe^3+^ is calculated between the differences of total iron minus the concentration of Fe^2+^. The calibration curve was built using FeSO_4_ standards.

## 3 Results

### 3.1 Fe^3+^ and Ni^2+^ supplementation enhance methane production during cometabolization of methanol and DCE by *M. barkeri*

**FIG 1:**
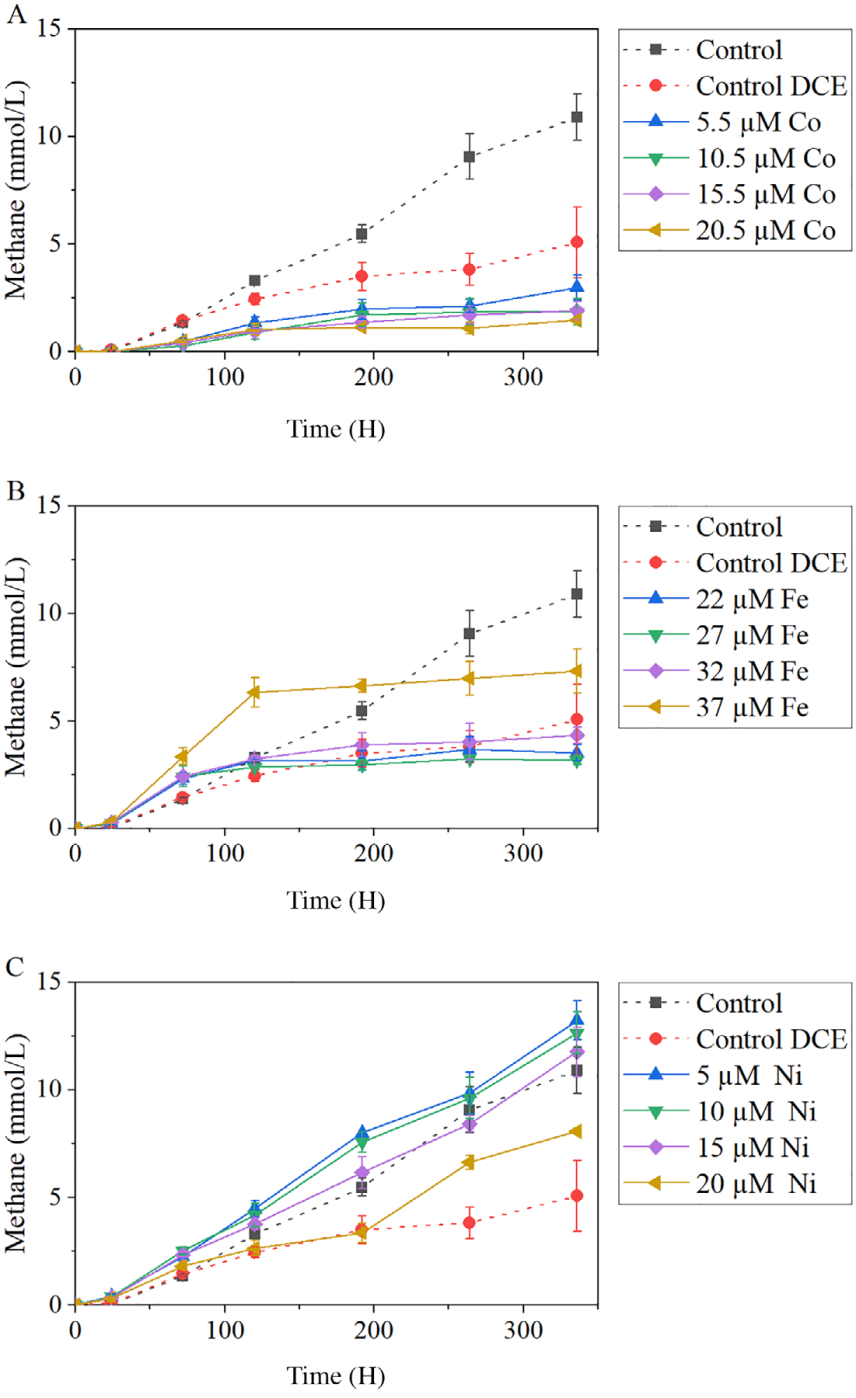
Cumulative methane production from the cometabolism of methanol and DCE by *M. barkeri*. Assays were done with supplementation of different concentrations of Co (A), Fe (B), and Ni (C); curves corresponding to controls “without DCE” (red circles) and “with DCE and no metals” (blue circles) are also shown.

Addition of 0.5 mM DCE to cultures of *M. barkeri* growing on methanol (125 mM) had an inhibitory effect on methane production; after 336 H of incubation methane production in assays with DCE was ∼50% less than in the controls without DCE (Fig. 1). Supplementation of *M. barkeri* cultures with Co^2+^, Fe^3+^, or Ni^2+^ was further on tested, resulting in distinct effect on methane production from methanol. Addition of Co^2+^ resulted in lesser methane from methanol, compared to cultures incubated with methanol and DCE without the supplementation of Co^2+^ (Fig. 1A). The lowest concentrations of Co^2+^ tested (5.5 µM) caused a decrease of about 40% in the cumulative methane produced after 336 H of incubation, when compared with the same conditions without metal. Higher Co^2+^ concentrations had an even higher inhibitory effect on *M. barkeri* and, for 20.5 µM Co^2+^, final methane production was only 70% of that measured in the control assay (Co‐Ctr). Supplementation of *M. barkeri* cultures with Fe^3+^ had a slightly beneficial effect on the initial methane production rates, with a maximum for the culture amended with 37 µM of Fe^3+^, that is, 0.81 ± 0.17 mmol CH_4_ day^−1^ (while 0.50 ± 0.12 mol CH_4_ day^−1^ was observed in the control with methanol and DCE). However, final cumulative methane produced was similar in controls with methanol and DCE and assays supplemented with Fe^3+^, except for the assay with 37 µM of Fe^3+^ that achieved substantially higher final cumulative production (i.e., 7.3 mmol/L compared to 5.01 mmol/L in control methanol plus DCE). Addition of Ni^2+^ resulted in the highest increase of both methane production rates and final cumulative methane concentration. Supplementation of 5–15 µM of Ni^2+^ resulted in a final cumulative methane production that was even higher than in control without DCE, and about 115–150% higher than in controls with DCE (Fig. 1C). Addition of 20 µM Ni^2+^ resulted in final higher methane production than in control with DCE, but lower than in cultures supplemented with 5–15 µM of Ni^2+^.

### 3.2 Trace metals affect hydrogen and acetate formation during cometabolization of methanol and DCE by *M. barkeri*

**FIG 2:**
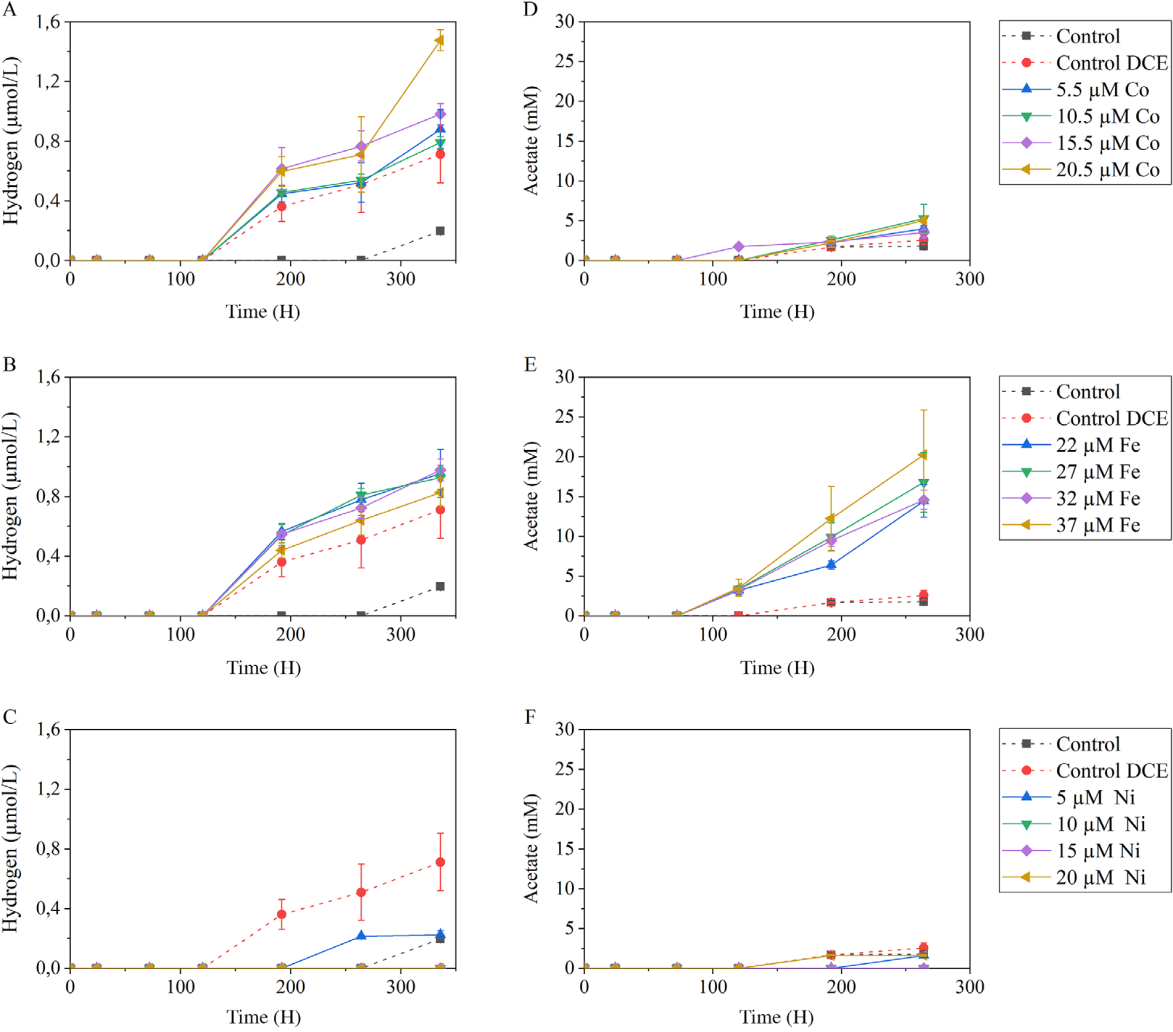
Cumulative hydrogen (A, B, C) and acetate (D, E, F) production in the presence of Co (A, D), Fe (B, E), and Ni (C, F).

Cultures of *M. barkeri* growing on methanol (control) produced mainly methane; accumulation of hydrogen and acetate by those cultures was negligible compared to assays with methanol plus DCE (control DCE) (Fig. 2). Hydrogen production by *M. barkeri* in cultures containing DCE was observed earlier (192 H) than in controls without DCE (355 H) (Fig. 2A). The initial Co^2+^ concentration affected the amount of hydrogen produced, with higher amounts of hydrogen measured for higher Co^2+^ concentrations (Fig. 2A). The acetate concentration (after 336 H) was approximately 45% higher in assays with DCE than in controls without DCE, but no major differences were observed for different Co^2+^ concentrations (Fig. 2D). Supplementation of 22, 27, and 32 µM of Fe^3+^ increased hydrogen production up to 60%, when compared to the control with DCE, whereas 37 µM of Fe^3+^ led to an increase of 25% hydrogen (Fig. 2B). Fe^3+^ amendment resulted in high acetate accumulation as well, for all the initial Fe^3+^ concentrations tested and up to about 20 mM in the assay with 37 µM Fe^3+^ (Fig. 2E). On the contrary to Co^2+^ and Fe^3+^ addition, supplementation of Ni^2+^ induced very little stimulation to hydrogen nor acetate production by *M. barkeri* from methanol (Figs. 2C and 2F).

### 3.3 *M. barkeri* is able to cometabolically reduce DCE to VC with methanol as electron donor and trace metals stimulate this conversion

**FIG 3:**
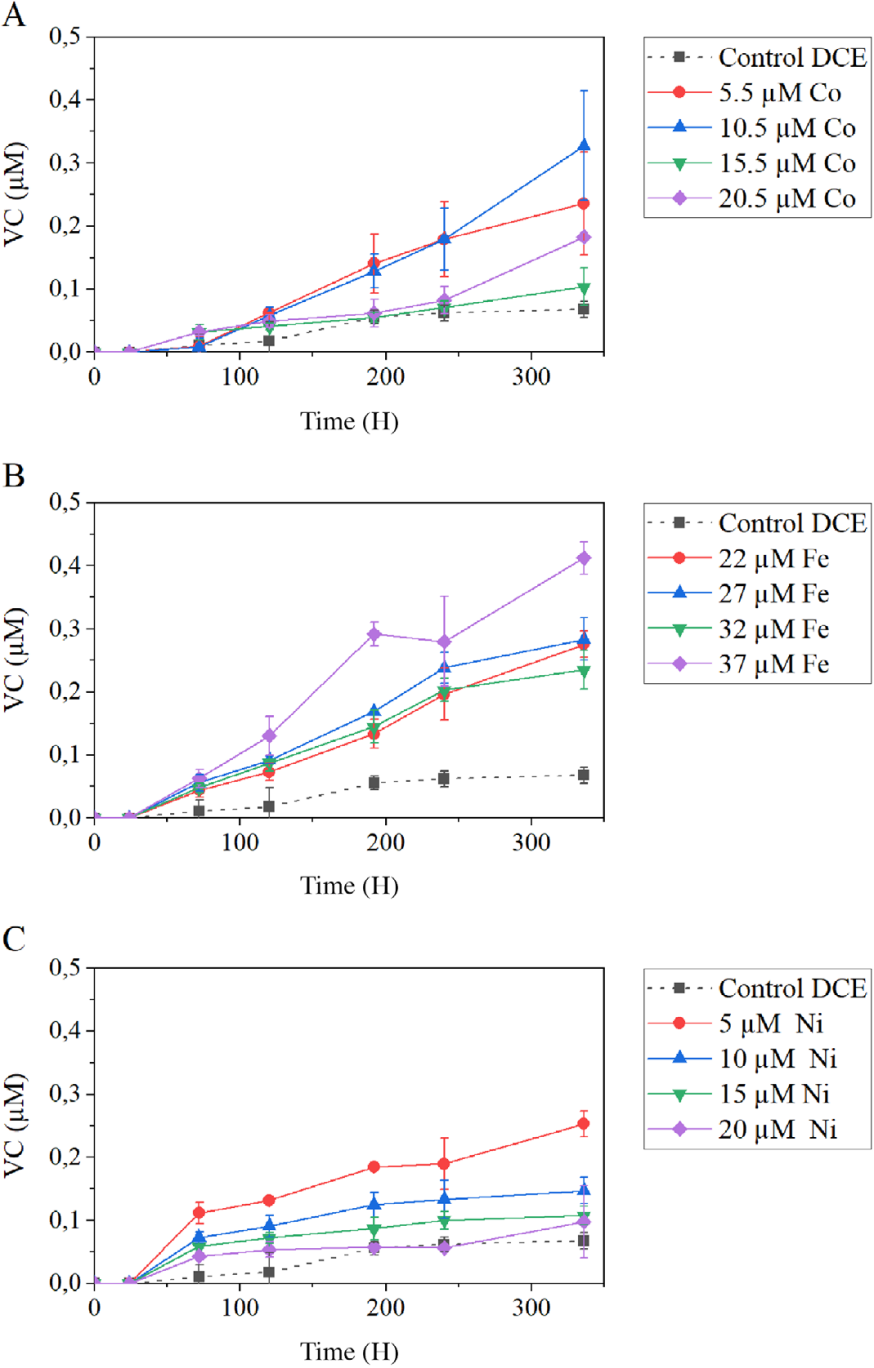
Cumulative VC production in the presence of Co (A), Fe (B), and Ni (C).

Supplementation of Co^2+^ to cultures of *M. barkeri* in the presence of DCE improved DCE reduction to VC (Fig. 3A). In the first 120 H of incubation, the effect of Co^2+^ was similar for all the concentrations tested. However, after that, 5.5 and 10.5 µM Co^2+^ had a stronger effect in the dechlorination rate and final VC formation in these assays was ∼250 and 380% higher than in controls, respectively. In the case of Fe^3+^ supplementation, 22, 27, and 32 µM of Fe^3+^ had similar positive effects on the formation of VC (Fig. 3B). 37 µM of Fe^3+^ had even a stronger effect and could improve the VC formation in over 500% compared to control assays. Amendment of Ni to *M. barkeri* cultures (with methanol + DCE) resulted in higher VC production than in control DCE (Fig. 3C). Highest VC production in Ni‐amended cultures was observed in assays with 5 µM of added Ni^2+^; higher Ni^2+^ concentrations resulted in less VC cumulative production. However, even for 5 µM of added Ni^2+^, the VC formed is less than when, for example, 37 µM of Fe^3+^ is supplemented.

### 3.4 Amendment of *M. barkeri* cultures with Fe^3+^ results in its reduction to Fe^2+^

It is known that *M. barkeri* is able to reduce iron Fe^3+^ [27, 28]. For this reason, the concentrations of Fe^3+^ and Fe^2+^ were measured at the end of the incubations with Fe^3+^ (Table 1). Most of the Fe measured in the final time point is in the form of Fe^2+^, indicating that there was iron reduction in the assays.

**Table 1:** Concentrations of total Fe, Fe^2+^, and Fe^3+^ (μM) at the end of the incubations with Fe and in the control with DCE

	Total Fe (μM)	Fe^2+^ (μM)	Fe^3+^ (μM)
Control DCE	2.24 ± 0.37	2.12 ± 0.29	0.12 ± 0.07
22 µM Fe	9.60 ± 1.20	8.01 ± 0.96	1.59 ± 0.24
27 µM Fe	12.76 ± 1.10	10.54 ± 0.88	2.22 ± 0.22
32 µM Fe	16.37 ± 0.81	13.43 ± 0.65	2.94 ± 0.16
37 µM Fe	24.15 ± 1.63	19.65 ± 1.31	4.50 ± 0.33

## 4 Discussion

Metal supplementation can be a good strategy to improve the performance of systems in which the reactions involve metal‐depending enzymes and/or cofactors. Our results show that both methane production from methanol as well as the cometabolic dechlorination of DCE by pure cultures of *M. barkeri* can be improved by metal amendment, but the effect depends on the metal ion and concentration. The tested *M. barkeri* (DSM 800^T^) was only able of partial dechlorination, producing VC from DCE. Mixed cultures in anaerobic digesters enclose high microbial diversity, and dechlorinating bacteria can aid in complete PCE/DCE dechlorination, as shown, for example, in the work by Heimann et al. [29]. The objective of the present study was, however, to study the effect of metals solely on methanogens, during cometabolic dechlorination and not evaluate their potential for complete dechlorination.

It has been shown before that the growth of *M. barkeri* is Co‐dependent and that Ni stimulated methanogenesis [30]. Cells of *M. barkeri* cultivated with methanol were reported to contain three times more corrinoids than cells grown with acetate [31]. In a study by Lin et al. [32], supplementation of 45 µM of Co^2+^, Fe^3+^, and Ni^2+^ enhanced the formation of corrinoids, cofactor F_430_, and cytochromes by *M. barkeri* cultures growing on methanol and about 70% of these compounds were excreted to the culture solution. However, it is not clear how metal supplementation affects cometabolic dechlorination. In this work, it was hypothesized that Co^2+^, Ni^2+^, and Fe^3+^ amendment could improve methane production from methanol and also the dechlorination of DCE. It was then observed that Co^2+^, Ni^2+^, and Fe^3+^ amendment can indeed help to improve DCE dechlorination: 20 µM of Fe^3+^ gave the best results increasing the VC formation by more than 500%, while for 5.5 µM of Co^2+^ and 5 µM of Ni^2+^ improved VC formation up to 380%. These results are most likely due to increase in the levels of the cofactors that are involved in the cometabolic dechlorination.

Methane production from methanol by *M. barkeri* was improved by Ni^2+^ supplementation, when compared with the control with DCE. However, 5 µM of Ni^2+^, the lowest tested Ni^2+^ concentration turned out as being the optimal concentration for both methanogenesis and reductive dechlorination. Increasing Ni^2+^ concentrations resulted in less cumulative methane production (Fig. 1C). Ni^2+^ supplementation has been previously shown to be beneficial for methanogens and anaerobic digestion [19, 33‐36]. On the other hand, amendment of Fe^3+^ increased the initial methane production rates, but final methane production was lower than in controls (Fig. 1B). Hydrogen and acetate production started after 72 H of incubation, simultaneously with the halt in methane production (Fig. 1B). The acetate final concentration in Fe‐supplemented cultures was 14–20 mM, while in controls only 2 mM of acetate were measured. At the end of the incubation of these assays, most of the Fe in the bottles was in the form of Fe^2+^. Inhibition of methane production due to iron reduction has been reported for *M. barkeri. Methanosaeta concilii*, and *Methanospirillum hungatei* [27, 28]. Growth of *M. barkeri* on 25 mM of methanol resulted in a higher degree of Fe^3+^ reduction than cultures growing on H_2_/CO_2_ [28]. In addition, the presence of Fe^3+^ inhibited methane production from methanol during the first 18 days of incubation, which is likely linked to the direction of electrons toward Fe^3+^ reduction instead of toward methane formation. Further studies revealed that *M. barkeri* rapidly switches from methanogenesis to iron reduction [37].

In the absence of H_2_, methanol is converted to methane according to Eq. (1). Three methyl groups are reduced to methane, and the fourth methyl group is oxidized to CO_2_ to produce the electrons needed for reduction of the other three methyl groups [38, 39].

(1) $$4CH_{3}\mathrm{OH}\;\to 3CH_{4}+1CO_{2}+2H_{2}O$$

Acetate production from methanol or H_2_/CO_2_ by *M. barkeri* was observed in the past; it was reported that *M. barkeri* could produce 30–75 µmol of acetate per mmol of CH_4_ formed [40]. Moreover, it was reported that *M. barkeri* growing on pyruvate produced acetate if bromoethanesulfonate, an inhibitor of methyl‐coenzyme M reductase, was added [41]. The authors proposed that pyruvate would be oxidized to acetyl‐CoA, which would then be converted to acetate via acetyl‐phosphate, coupled to ATP production.

The addition of Co^2+^ had a negative effect on methane production by *M. barkeri*, which was not expected because methyl‐cobalamin is directly involved in methane production. However, similar inhibitory results were previously observed in cultures of *M. barkeri* growing on methanol and supplemented with 5 µM of Co^2+^ [42]. However, hydrogen production was enhanced by Co^2+^ supplementation and acetate production also increased slightly. Furthermore, cometabolic dechlorination was also enhanced by Co^2+^ addition. In the methylotrophic pathway, Co^2+^ is required for methanol‐coenzyme M methyltransferase and CH_3_‐H_4_M(S)PT‐coenzyme M methyltransferase, the first enzymes in the reductive and oxidative branches, respectively. Our results seem to indicate that Co^2+^ amendment increases the levels of cofactors available, which leads to an increase of the dechlorination rates. However, this is not reflected in an increase of methane production, which can be due to limitations of the levels of other metal ions, such as Ni, that are required for other enzymes of the pathway.

In conclusion, our results show that metal ions can be a limiting factor in methanogenesis and dechlorination that require metal‐depending enzymes or cofactors and that metal supplementation can lead to considerable improvement. However, the beneficial effect depends on the metal and concentration added, as well as which of the processes is intended to be enhanced. If applied the right conditions and dosages, metal amendment represents a promising strategy to enhance biogas production and mitigate the toxic effects of chlorinated compounds.

## 5 Authors’ contribution

LMP and DZS designed the experiments. LMP performed the experiments with the help from MRH and GM. LMP analyzed the data and drafted the manuscript. DZS and AJMS critically revised the manuscript. All authors read and approved the manuscript.

## 6 Conflict of Interest

Authors declare no conflict of interests.

## Supporting information

Supplementary Material

Figure S1 ‐ Example of methane production curve in a batch assay with 37 µM of Fe. Circles with different colours indicate results from 3 independent replicas, and triangles represent the average values. Bars represent standard deviation.
