# Supplementary material for: Effect of nickel, cobalt, and iron on methanogenesis from methanol and cometabolic conversion of 1,2‐dichloroethene by Methanosarcina barkeri
Source: Biotechnol Appl Biochem. 2020 May 12;67(5):744–50. doi: 10.1002/bab.1925 (PMC7687089; doi:10.1002/bab.1925)
Supplement: Supplementary file 1 — Supplementary Material [file BAB-67-744-s001.pdf]

Effect of nickel, cobalt and iron on methanogenesis from methanol and co-metabolic conversion of 1,2-dichloroethene (DCE) by *Methanosarcina barkeri*

Lara M. Paulo[1], Mohamad R Hidayat[1], Giulio Moretti[1,2], Alfons J. Stams[1], Diana Z. Sousa[1]

[1]Laboratory of Microbiology, Wageningen University & Research, The Netherlands
[2]Laboratory of Microbiology, MESVA Department, University of L'Aquila, Italy

Supplementary Material

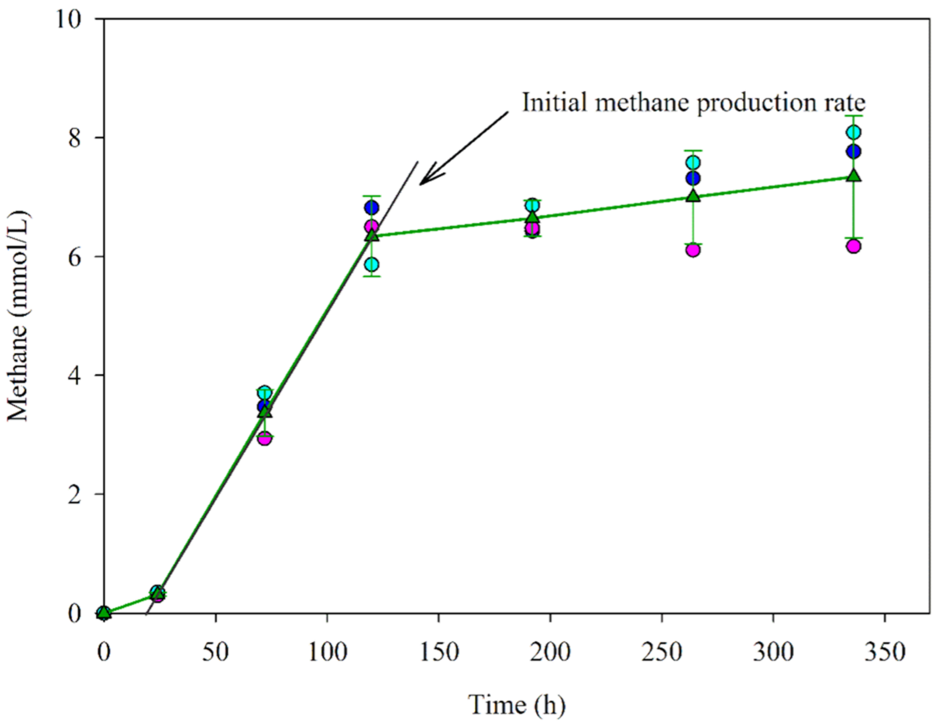

Figure S1 - Example of methane production curve in a batch assay with 37 µM of Fe. Circles with different colours indicate results from 3 independent replicas, and triangles represent the average values. Bars represent standard deviation.
