# Supplementary figures and images for: Effect of nickel, cobalt, and iron on methanogenesis from methanol and cometabolic conversion of 1,2‐dichloroethene by Methanosarcina barkeri
Source: Biotechnol Appl Biochem. 2020 May 12;67(5):744–50. doi: 10.1002/bab.1925 (PMC7687089; doi:10.1002/bab.1925)

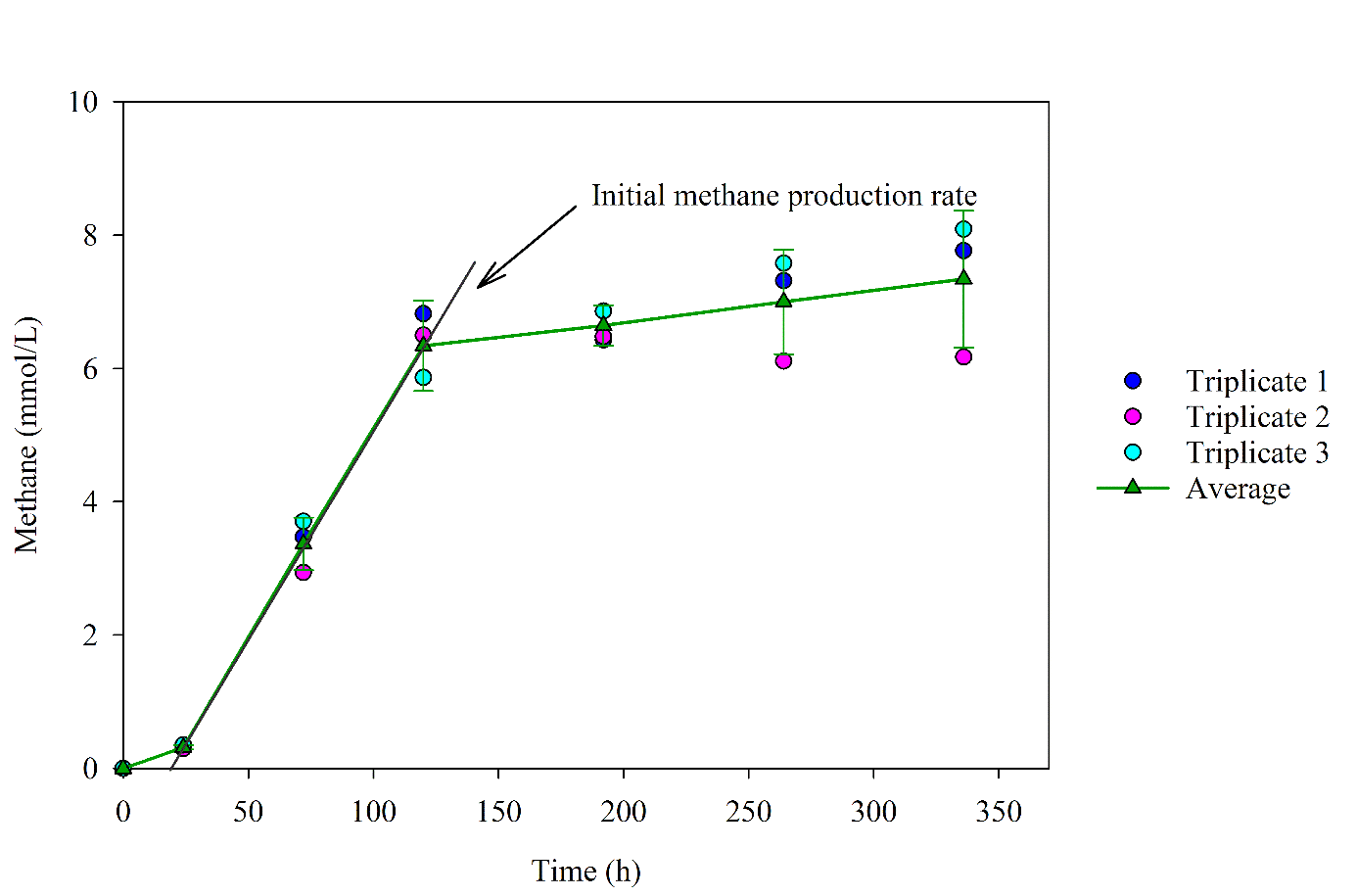

Supplement: Supplementary file 2 — Figure S1 ‐ Example of methane production curve in a batch assay with 37 µM of Fe. Circles with different colours indicate results from 3 independent replicas, and triangles represent the average values. Bars represent standard deviation. [file BAB-67-744-s002.tif]
